# Respiratory Allergens from Furred Mammals: Environmental and Occupational Exposure

**DOI:** 10.3390/vetsci4030038

**Published:** 2017-08-04

**Authors:** Eva Zahradnik, Monika Raulf

**Affiliations:** Center of Allergology/Immunology, Institute for Prevention and Occupational Medicine of the German Social Accident Insurance, Institute of the Ruhr-Universität Bochum (IPA), Bürkle-de-la-Camp-Platz 1, 44789 Bochum, Germany; raulf@ipa-dguv.de

**Keywords:** animal allergens, allergen exposure, cat, cattle, dog, horse, mouse, rat

## Abstract

Furry mammals kept as pets, farm and laboratory animals are important allergen sources. The prevalence of sensitization to furred mammals appears to be increasing worldwide. Several mammalian allergens from diverse species are well characterized with regard to their molecular structure and immunogenicity, and some are already available for component-resolved allergy diagnostics. The distribution of various mammalian allergens has been extensively studied during the past few decades. Animal allergens were found to be ubiquitous in the human environment, even in places where no animals reside, with concentrations differing considerably between locations and geographical regions. This review presents an overview of identified mammalian respiratory allergens classified according to protein families, and compiles the results of allergen exposure assessment studies conducted in different public and occupational environments.

## 1. Introduction

**Figure 1:**
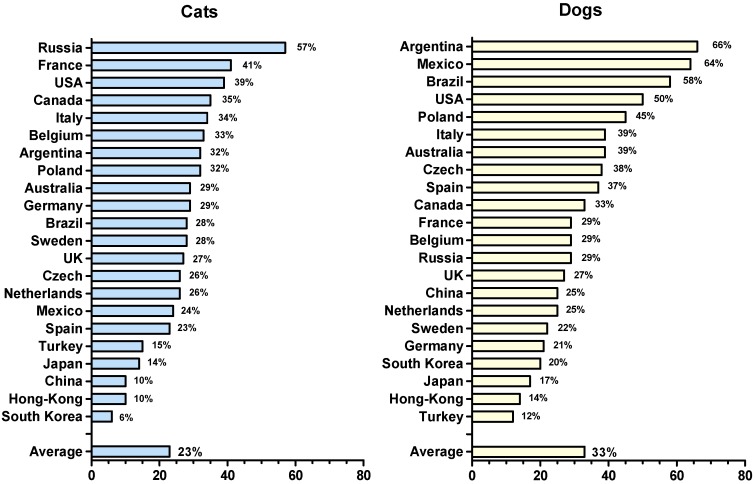
Frequency of cat and dog ownership in different countries **(**based on Global GfK survey [3]).

Exposure to allergens is a relevant risk factor for the development of sensitization and respiratory allergic diseases, such as asthma and rhino-conjunctivitis [1,2]. Besides pollen and house dust mites, furry animals are among the most common sources of respiratory allergens. Domesticated mammals typically kept as pets, farm or laboratory animals play an important role in human life. They serve as companions, resources, workers, and research objects. According to an online survey of more than 27,000 people across 22 countries conducted in 2015 by the German market research company (Gesellschaft für Konsumforschung; GfK), more than half (56 percent) of the international population have at least one pet (including dogs, cats, birds, fish and others) at home [3]. Cats and dogs are most common, and the frequency of their ownership is highly variable, averaging 33% for dogs and 23% for cats worldwide (Figure 1) [3]. According to research by the Worldwatch Institute, there is a global trend of rising farm animal populations due to increasing demands for meat, eggs, and dairy products, especially in developing countries. For example, between 1970 and 2010 the cattle population grew 32 percent to reach 1.4 billion, and is projected to continually increase to nearly 2.6 billion animals by 2050 [4]. There is also an increase in the use of laboratory animals for medical and life-science research. In Great Britain, for instance, the number of procedures using mice doubled from about 1.5 to 3 million in the period 1995–2012, with a sharp rise in the number of genetically modified animals in recent years [5].

The prevalence of allergic sensitization to mammalian animals varies in different countries according to cultural differences, environmental factors, and rate of pet ownership [6]. The geographical variation in the prevalence of positive skin tests to diverse environmental allergens has been shown by Bousquet et al. in a large multicenter study (about 15,000 subjects living in 35 centers in 15 countries) [7]. The rate of sensitization to cats was 8.8%, ranging from 1.2% to 22.4%. Of course, in patient populations, the sensitization frequencies are higher compared with those detected in the general population. A large patient-based study of “The Global Asthma and Allergy European Network” (GA^2^LEN) found that 26% and 27% of the participants were sensitized to cats and dogs, respectively [8]. Several population-based studies from different countries have shown that over the past decades the prevalence of sensitization has been increasing in both adults and children [9,10,11,12,13]. For example, a study conducted in Sweden has shown an increase in sensitization to cat (16% to 26%), dog (13% to 25%), and horse (8% to 10%) from 1994 to 2009 [14].

Mammalian allergens are present mainly in skin, saliva and urine of animals. These allergens stick to fur and are efficiently dispersed into the environment as animals shed hair and dander, and secrete and excrete bodily fluids. In addition, these allergens tend to be carried on small dust particles that remain airborne for long periods of time after disturbance. Based on their aerodynamic properties, mammalian allergens attach to human clothing and hair [15,16,17], and can be easily transferred to environments that were never previously occupied by the animal. Once indoors, the allergens accumulate primarily on different textiles, such as carpets, upholstered furniture and mattresses where most likely due to their high stability, they remain detectable for a long time. 

In general, mammalian allergens are ubiquitous in the human environment, even though concentrations differ considerably [18]. Most studies that have examined exposure to animal allergens have focused on the home environment, where the allergen concentrations are primarily dependent on the presence of animals. Cat and dog allergen levels were found to be approximately 50-fold to 200-fold higher in homes with pets than in homes without pets [19,20,21]. Over the past decades, nonresidential indoor environments have also been recognized as an important site of exposure to animal allergens. This observation is primarily due to the large amount of time that both adults and children spend outside the home, for example, at work, school or in day care centers. Whereas the level of exposure in the home can be reduced to some degree by the residents themselves (e.g., by pet removal or cleaning), the avoidance of allergens in locations outside the home is often not possible. Therefore, the purpose of this article is to summarize the results of allergen exposure assessment studies conducted in various public and occupational indoor environments. In addition, an overview of identified mammalian respiratory allergens is also included.

### 1.1. Allergens

An allergen is any substance that elicits an immunoglobulin E (IgE) antibody response. During the past few decades, several animal allergens have been identified and characterized at the molecular level. To date, 35 mammalian allergens are officially recognized by the subcommittee of the World Health Organization/International Union of Immunological Societies (WHO/IUIS), and are listed in the allergen database, www.allergen.org (Table 1). The designation of allergen names is derived from the allergen source: the first three letters of the genus, the first letter of the species, and a number that indicates the chronology of the allergen’s discovery (e.g., Fel d 1, the first allergen from the cat *Felis domesticus*). 

All major allergens from mammals can be classified into four protein families: lipocalins, secretoglobins, kallikreins and latherins [22]. Officially, a major allergen is an allergen that is recognized by IgE antibodies in >50% of patients allergic to the allergen source [23]. In most cases, major allergens bind a large fraction of the allergen-source specific IgE, and are (most likely) of major clinical importance. Another large protein family among animal allergens is represented by serum albumins [24,25], which are present in the blood of animals where they regulate osmotic pressure and transport diverse molecules. Albumins are highly conserved both in amino acid sequence and three-dimensional structure and have a molecular weight of 65–69 kDa [24]. Serum albumins are also present in animal dander, milk and other secretions and are considered to be minor allergens, because sensitization rates are up to 30% in patients allergic to furred mammals [25]. Nevertheless, albumins are relevant because they are often responsible for allergic cross-reactions between animal dander of different species due to the high sequence identity (72–82%) [24].

An important part of allergy diagnosis is based on specific IgE detection using allergen extracts obtained from materials, such as cat dander or fur. Indeed, the crude allergen extracts imitate the source of natural exposure, but on the other hand they are difficult to standardize due to the heterogeneity of raw materials. In some cases, allergens may be absent or underrepresented in the manufactured extract. Progress in molecular biology over the last 30 years has permitted the production of proteins in their highly purified native or recombinant form. The use of single allergenic components allows for the improvement of assay sensitivity and specificity, increased knowledge of sensitization profiles and cross-reactivity, and the determination of markers for species-specific sensitization. In recent years, the number of publications using the so called component-resolved diagnostics (CDR) is increasing. Unfortunately, the number of commercially available components is still limited. Several major and minor allergens are available for cats and dogs, but no molecules exist for other small pets such as guinea pig, hamster or rabbit. Mammalian allergens currently available for CDR are marked with an asterisk in Table 1.

### 1.2. Lipocalins

Lipocalins make up the majority (>50%) of the mammalian respiratory allergens. At least one major lipocalin allergen has been identified in each species, for instance: Can f 1 in dogs, Fel d 4 in cats, Equ c 1 in horses, Bos d 2 in cattle, and Mus m 1 and Rat n 1 in rodents (Table 1). The name, lipocalin, is derived from two Greek words: lipos and kalyx. It reveals the most widely recognized feature of these proteins: its binding to various lipophilic molecules [44]. Lipocalins are small, secreted molecules of around 200 amino acids, with molecular weight averaging 20 kDa. Although the sequence identity among the lipocalins is low (usually between 20 and 30%), this protein family is characterized by a common tertiary structure that contains an internal hydrophobic binding pocket. In general, mammalian lipocalin allergens are predominantly considered to be carrier of odorants and pheromones. However, their function is largely unknown [45]. Apart from the transport of molecules, lipocalins are involved in stress and inflammatory reactions, as well as in the pathways of signal transduction [44]. 

Lipocalin allergens are mainly produced in the liver or secretory glands, and localized in animal skin and body fluids, such as urine, saliva, and sweat or sebum. Due to the low degree of sequence identity among them, lipocalins were considered as species-specific allergy markers. However, some lipocalins do display a higher degree of sequence similarity (up to 67%), and cross-reactivity can occur as shown for Fel d 4, Equ c 1 and Can f 6 [46,47], Mus m 1 and Rat n 1 [48], Can f 1 and Can f 2 [34], and Equ c 1 and Mus m 1 [34].

### 1.3. Secretoglobins

Thus far, only two mammalian allergens have been categorized as members of the secretoglobin protein family: Fel d 1 from cat and Ory c 3 from rabbit. Fel d 1 is the most extensively studied animal allergen with regard to its molecular structure, IgE reactivity, T-cell responses, aerodynamic properties, environmental distribution, and the relationship between allergen exposure and the development of allergic disease. The biochemical characterization of Fel d 1 and its role in diagnosis and immunotherapy has been extensively summarized by Grönlund et al. [49]. Fel d 1 is produced in sebaceous, anal, and salivary glands, and is mainly present in skin and on fur. It is a tetrameric glycoprotein formed by two 18 kDa non-covalently linked heterodimers. Despite intensive research, the biological function of this major allergen remains unknown since its discovery 4 decades ago [50]. The role of Fel d 1 in cat allergy is very dominant, because it reacts with IgE from 90% of cat-sensitized individuals (as shown in different patient collectives), and it accounts for 60–90% of the total reactivity to cat dander [26,51]. The second secretoglobin, Ory c 3 has been identified in rabbit fur. Similar to Fel d 1, it consists of two chains forming a heterodimer and is reported as a major allergen because of an IgE binding frequency of 77% in rabbit allergic patients [43].

### 1.4. Kallikrein

At present, Can f 5 is the only animal allergen belonging to the kallikrein family. Can f 5 was isolated from the urine of male dogs and identified as a prostatic kallikrein [33]. Can f 5 was also shown to be present in dog dander, and its amino acid sequence shows no significant similarity to any known animal dander or urinary allergen. It is considered a major allergen because 70% of patients with dog allergy showed IgE reactivity to recombinant Can f 5, and 38% of these patients reacted to this protein alone [33]. Subsequent studies have found much lower sensitization rates (31–46%), but with similar high proportion of Can f 5 monosensitized patients [27,29,30].

### 1.5. Latherin

Latherin, later named Equ c 4, is a major component of horse sweat and has strong surfactant properties [52]. Equ c 4 has also been found in horse saliva [53], and its three-dimensional molecular structure and proposed method of action has been recently reported by Vance et al. [54]. Latherin probably acts as a wetting agent to facilitate evaporative cooling through a waterproof pelt. The detergent-like activity of Equ c 4 likely causes foam formation on the pelt of sweating horses, especially where rubbing occurs. To date, the IgE binding frequency to Equ c 4 in 77% of horse sensitized subjects has been only reported by one study [35]. To evaluate this result, further investigations are needed. Fel d 8 protein, a latherin-like allergen has also been characterized from the salivary glands of cats [55]. However, with an IgE binding frequency of only approximately 20%, Fel d 8 is considered a minor cat allergen.

## 2. Measurements of Allergen Exposure 

Assessment of allergen exposure levels is a stepwise process that involves dust collection, allergen quantification and data analysis. For each step, many alternative methods are available. Several dust sampling collection procedures have been described in the literature, including reservoir dust sampling using a vacuum cleaner, air sampling with filters using person-carried or stationary pumps, and sampling of airborne settled dust using petri dishes or electrostatic cloths [56,57,58]. Apart from the type of dust, the collection methods can also introduce variability in the results due to differences in sampling equipment, size and surface of the area sampled, sampling duration, and sampling location. The choice of sampling method is often influenced by the type of environment to be examined, the size of the study, available budget, practical performance, and relevance to the personal exposure. 

Compared to dust sampling strategies, allergen quantification methods used in studies are more uniform. Almost all measurements of exposure to mammalian allergens are based on the estimation of major allergens for each species using immunoassays such as enzyme linked immunosorbent assay (ELISA) or multiplex array for indoor allergens (MARIA). The latter method allows for the simultaneous measurement of 6–10 allergens in a single test. Occasionally, the measurements are performed using assays against extracts that contain several allergenic components (e.g., cow hair extract or mouse urine). The variations between the different immunoassays are usually dependent upon the type of antibodies, calibration standard and its protein determination, and detection/visualization methods. The EAACI Position Paper from 2014 includes an extensive summary of the different methods used for sampling and allergen quantification, as well as their pros and cons in various exposure settings [58]. Importantly, the methodological differences among studies enormously influence the results of exposure measurements. Furthermore, the resulting allergen concentrations are not directly comparable because of different units, and the variability in results that are at times several orders of magnitude. Nevertheless, many studies from different exposure settings show similar effects regarding the factors that influence allergen levels.

## 3. Animal Allergens in Occupational Settings

### 3.1. Laboratory Animal Facilities

Mice and rats are by far the most widely used animals in medical research. Therefore, numerous studies investigating mouse and rat allergen exposure levels were carried out in laboratory animal facilities at universities, research institutes, and pharmaceutical companies. The sampling method commonly used in these occupational settings is collection of airborne dust using pumps. The allergen concentrations can vary strongly between the individual facilities. For example, one study measuring Rat n 1 levels in 12 different rat facilities in France reported a range of concentration from 0.49–48.96 ng/m^3^ in personal airborne samples, and 0.43–27.36 ng/m^3^ in stationary airborne samples (the values represent the geometric means (GM) of all collected samples in the 12 sites) [59]. Within one facility, the allergen concentrations were found to be dependent on several factors, such as the type of room, number of animals, and activities performed in the room. Several studies have shown that rodent allergens are widely distributed throughout the facility, even in rooms where no animals are present, such as offices, staff rooms, corridors and laboratories [60,61,62], although the concentrations are often much lower compared to rooms where animals are housed. For example, in one mouse breeding facility Mus m 1 values ranged from 17 to 564 ng/m^3^ in the mouse rooms, and from 1.2 to 2.7 ng/m^3^ in offices [60]. In another study, significantly higher mean Rat n 1 concentrations were measured in rat rooms (53.1 ng/m^3^) than in experimental rooms (9.7 ng/m^3^) [59]. In the animal holding rooms, allergen concentrations increased with the number of animals [63] and declined with increasing relative humidity [64]. In addition, the type of cage system used in the animal rooms was shown to have a considerable influence on the allergen concentrations [62,65]. Allergen concentrations were significantly lower in rooms with individually ventilated cages (IVC) compared to rooms with open cages (median Mus m 1: 4.3 vs. 44ng/m^3^ [65]). Moreover, the IVCs are more effective at reducing allergen levels when operating in the negative rather than positive pressure mode. 

Further studies examined the personal allergen exposure intensity according to the type of job and the type tasks performed. The highest allergen exposures were observed in animal technicians and caretakers compared to scientists and students [59,61,66]. Cage cleaning resulted in much higher allergen levels than animal handling (mean Rat n 1: 91.1 ng/m³ vs. 5.4 ng/m³ [59]), and animal care was associated with higher allergen concentrations than performing laboratory experiments (median Mus m 1: 8.73 ng/m³ vs. 0.36 ng/m³ [67]). Allergen exposure can also occur in persons who have no contact to animals. In a mouse research and production facility, Mus m 1 median concentrations were 0.23 ng/m³ among the administrative/support personnel and 0.63 ng/m³ among the materials/supplies handlers [67]. Several practices have been found to reduce the concentrations of rodent allergens in animal rooms. For instance, the use of cage-changing stations reduced the concentrations of mouse or rat allergens in the handler’s breathing zone by 80–95% [62,68,69]. The use of a vacuum bedding-disposal system was also shown to reduce median personnel exposures 25-fold compared to conventional waste containers [62], and the installation of special polycarbonate curtains in front of the cages even led to non-detectable allergen concentrations [70].

Rodent allergens can be transferred from the facilities to homes. Krop et al. investigated mattress dust from laboratory animal workers and non-exposed controls and found significantly increased mouse and rat urinary allergen levels (median RUA: 39.3 vs. 7.6 ng/g, median MUA: 29.5 vs. 8.8 ng/g) [71]. The allergens are most likely transported via uncovered hair into the living area, because in contrast to daily changes in work clothing, hair-covering caps are not routinely worn by laboratory animal workers.

### 3.2. Animal Farms

In recent years, several exposure measurements have been carried out in stables and homes of cattle farmers. In cow stables, levels of bovine allergen were estimated using different dust sampling methods. Virtanen et al. reported airborne concentrations of bovine allergens with a mean of 430 ng/m³ in personal measurements and between 350 ng/m³ (feeding passage) and 750 ng/m³ (manure passage) in stationary measurements [72]. The median concentration of major bovine allergen Bos d 2 in the reservoir dust from stables was 20,400 µg/g [73], and the median concentration of cow hair allergens in dust collected with electrostatic dust fall collectors (EDC) was 51,700 µg/m² [74]. EDC measurements also indicated slightly higher levels of bovine allergens in summer (GM 45,400 µg/m²) compared to winter (GM 37,100 µg/m²) [75]. Stables housing mainly calves showed borderline lower bovine allergen levels compared with levels in main stables with lactating cows [75]. In all studies, the allergen levels were highly variable (up to 200-fold) among the individual stables. 

Due to the passive transport of allergens through workwear, bovine allergens are also detectable in the homes of farmers sometimes at relatively high concentrations [73,74,75], although these levels are still much lower than those measured in the stables. In homes of Bavarian cattle farmers suffering from occupational asthma or rhinitis due to bovine dander, Bos d 2 levels in mattress dust (median: 195 µg/g) and living room floor dust (median: 155 µg/g) were 100 times lower than in the cattle stables [73]. In addition, two studies using EDC to investigate settled airborne dust reported concentrations of cow hair allergens that were 5000 times lower in the farmers’ bedrooms than in the cattle stables (median: 11.8 µg/m² [74] and GM: 8.3 µg/m² [75]). The different factors (100 vs. 5000) may be due to the difference in the dust sampling method. The sampling time of the EDC was limited to 2 weeks, but allergens can accumulate in reservoir dust over longer periods of time. A recent study has shown that there is a steep concentration gradient in bovine hair allergens from stables to the dwelling areas of farmers. The highest median concentrations were reported in the milking parlor (7154 µg/g), followed by decreasing concentration in the computer room in the stable (2165 µg/g), the changing room (380 µg/g), the living room (109 µg/g), and finally the bedroom (63 µg/g) [76]. In this study, the authors showed that changing and private rooms at farms with automatic milking systems were less contaminated with bovine allergens than farms with conventional milking systems. One possible explanation is that there is less direct contact with the animals leading to lower allergen exposure and transfer. Interestingly, bovine allergens were still detectable in homes of farmers who had given up cattle husbandry for at least 2 years. Terminating work in cattle stables reduced Bos d 2 levels in both the living rooms and in mattress dust by about 20 fold compared to dust samples collected from farmers with regular contact to cattle (living room: 13 µg/g vs. 316 µg/g; bedroom: 12 µg/g vs. 265 µg/g) [73]. Finally, the distribution of cattle allergens was assessed at different distances to dairy farms in the Yakima Valley, Washington, USA, where over 60 industrial scale dairies operate [77]. In this study, indoor and outdoor settled dust was collected from 40 homes that varied in their proximity to the dairies. A concentration gradient of Bos d 2 was especially apparent in the outdoor samples collected from proximal (within a 1/4 mile, 0.4 km) to distal homes (>3 miles, 4.8 km). Bos d 2 was detected in 79%, 57%, and 23% of proximal, intermediate, and distal homes, respectively. The median Bos d 2 concentrations in the proximal homes were 17 times higher than in the distal homes.

In addition to cattle farms, allergen measurements were performed in horse stables and their immediate surroundings [78,79,80]. In general, outdoor horse allergen levels declined rapidly with increasing distance from the stable. Airborne levels of the horse allergen, Equ c 4 were more than 500-fold higher inside the stable (439,000 U/m³) than at the stable entrance (1140 U/m³), and more than 3000-fold higher than at a residential building located only 12 m from the stable (150 U/m³). Horse allergens could not be detected in air samples collected 40 m from the stable [78], which was supported in a later study by Elfman et al. [79]. The authors reported that horse allergen generally spread in ambient air approximately 50 m outside the stable and outdoor area where horses are kept, such as pastures or riding grounds. Depending on wind speed and direction, low levels of allergen (2–4 U/m³) were occasionally found in open areas between 300 and 500 m from the stable. Allergen levels were also found to be influenced by season. Here, the median Equ c 4 levels measured at the pastures in autumn (20 U/m³) were half those measured in summer (43 U/m³), and winter levels (9 U/m³) were a quarter those measured in summer. The median level at the entrance of the stable was 316 U/m³ in summer and 123 U/m³ in winter. Possible reasons for the reduced spread of allergen could be the increased rainfall in autumn, and covering of the horses in winter. Horse allergen was also detected in indoor (6 of 45) and outdoor samples (16 out of 26) that were collected using Petri dishes from homes located up to 250 m from stables [80]. To date, there are no studies on horse allergen levels in homes of occupationally-exposed persons or those exposed from leisure activities. 

### 3.3. Veterinary Clinic

Apart from laboratory animal workers and farmers, veterinarians are among those who are most exposed to animal allergens. Most veterinarians (and co-workers) work in private medical practices, where exposure levels to animal allergens are mostly unknown. Thus far, there is only one study that characterized cat and dog allergen concentrations in a companion animal hospital using various dust collection methods (airborne dust, EDC, vacuumed floor dust and table wipe dust) [81]. Here, the authors showed that allergen levels varied greatly between the performed tasks and the different locations. In general, Can f 1 was detected more often and at much higher levels when compared to Fel d 1, because dogs represented 85% of all animals treated during the sampling period. In personal airborne samples, the highest exposure was observed in the intensive care unit (Fel d 1, GM 1.5 ng/m³; Can f 1, GM 18.8 ng/m³), and the lowest in the operation room (Can f 1, GM 0.6 ng/m³) and anesthesiology room (Fel d 1, GM 0.1 ng/m³), most likely due to the more regular and intensive cleaning, and less contact with active animals. In the floor dust samples, the highest allergen levels were measured in examination rooms (Fel d 1, GM 39 ng/m^2^; Can f 1, GM 1148 ng/m^2^) and waiting rooms (Fel d 1, GM 36 ng/m^2^; Can f 1, GM 2101 ng/m^2^). This study also showed that often-used surfaces, such as examination tables for animals (Can f 1, GM 779 ng/m^2^), computer desks (Can f 1, GM 512 ng/m^2^), and tables for equipment (Can f 1, GM 118 ng/m^2^) can also be potential sources of allergens.

## 4. Animal Allergens in Public Places

### 4.1. Schools and Day Care Centers

Schools and day care centers, where children and teachers spend a large part of their time, may also be important sites of exposure to animal allergens, particularly for susceptible individuals. A comprehensive compilation of 35 studies conducted in educational facilities worldwide has been published by Salo et al. in 2009 [82]. The authors summarized the key findings from the scientific literature to date. The most important were: (1) the number of pet owners at school or daycare centers is one of the strongest predictors of elevated cat and dog allergen levels in these settings; (2) allergen levels in educational facilities can sometimes be significantly higher than in the home environment; (3) allergen levels can vary in different parts of the world depending on a variety of geographic, climatic and cultural factors; and (4) carpeting, upholstered furnishings, and clothing are important reservoirs for allergens. These findings were also confirmed in recent studies focusing on schools and day care centers [83,84,85,86,87,88,89]. The results from these studies are presented in Table 2 that includes information on sampling location, dust sampling method, allergen concentrations and percentage of positive samples.

An investigation into the presence of horse allergen in Swedish schools revealed that allergen levels were higher in classrooms containing higher proportion of children who had regular contact with horses in their leisure time [83]. This result strongly suggests that transfer of allergens in animal-free environments takes place via contaminated clothing. Using two different dust collection methods (vacuumed dust and EDC), two recent studies from the United States [84] and the Netherlands [85] have found higher cat, dog and mouse allergen levels in classrooms compared to the homes of students, especially those without pets. As shown in the Dutch study, there was a 10-fold difference in Fel d 1 and Can f 1 levels between schools and homes without the respective animal. The significant indicator for cat and dog allergens in classrooms and homes was the presence of children who had pets at their home (results of multivariate analyses). In addition, allergen levels in classrooms were influenced by the socioeconomic status of the children. The comparison of these two studies is also a good example of the geographic variation of some allergens. Levels of mouse allergen found in the U.S. study in both home and school environments were significantly higher than the reported cat or dog allergen levels. In the Dutch study, the amount of mouse allergens was very low. Mus m 1 is widely distributed in U.S. communities and is commonly found in almost all inner-city homes in the northeastern United States [90,91]. However, in Europe the exposure to mouse allergen in the home environment seems to be very low (about 50 times lower) [92]. In contrast to pet allergens, it is unlikely that the mouse allergen is brought into schools via contaminated clothing. Rather, the elevated levels of Mus m 1 in schools are indicative of rodent infestations at the schools and not the passive transfer from students’ homes. Permaul et al. found a relationship between signs of mice (visible mouse droppings) and higher levels of mouse allergen [86]. Compared with mouse allergen, the rat allergen, Rat n 1 is hardly detectable in both the United States and Europe [84,92,93], suggesting that rat allergen exposure may occur primarily outdoors. The difference in prevalence of rat and mouse allergens is consistent with the behaviors of these two rodents, as rats tend to be outdoor dwellers and mice prefer an indoor environment. 

A recent study from Malaysia provides another example of the variability of allergens in different parts of the world. Cat, dog and horse allergens were not detectable in schools [87], suggesting relatively low levels of these allergens in tropical countries as shown previously for educational facilities in Brazil [94] and Singapore [95]. Furthermore, the distribution of Can f 1 and Fel d 1 in German day care centers was shown to differ according to the sampling site and vacuumed surface [89]. Higher allergen concentrations were found in furniture compared to carpets; and carpeted floors contained higher allergen levels than smooth floors. There was a significant correlation between allergen concentrations on the different surface types that were sampled in the same room at the same time. A recent study from Poland showed that children’s clothing is the most important reservoir for animal allergens [88]. Very high concentrations of Can f 1 and Fel d 1 were found in the dust vacuumed from the clothes of children who had a dog and/or cat at home. In the same study, both cat and dog allergen levels were found to be higher in settled dust that was collected from the floors of kindergartens in winter, compared to samples collected in the same rooms in summer, although the difference was only statistically significant for Can f 1. One explanation for the seasonal variation may be the clothing type worn in different seasons, as woolen sweaters and jackets have been shown to contain higher allergen levels than T-shirts [16].

### 4.2. Public Buildings

In contrast to the extensively investigated school and daycare center environment, there are few studies on animal allergen exposure in public buildings (Table 2). The presence of cat and dog allergens in different public places was shown in the 90s by Custovic et al., and included 5 schools, 6 hotels, 4 cinemas, 6 pubs, 3 buses and 2 trains [96,97]. Major allergens from both species were found in all samples. In general, allergen levels were significantly higher in the dust from upholstered seats compared to those collected from carpeted floors, presumably because they came into direct contact with clothing. The same was found upon comparing the allergen concentrations between carpets and seats in each of the public places. Both these sampling sites in public places had higher Fel d 1 and Can f 1 levels than private homes without the respective animals. The authors concluded that upholstered seats in public places constitute an allergen reservoir for continuous contamination of the indoor environment, which could compromise the effects of allergen avoidance employed at home. 

Some recent studies have limited their research to a specific type of public building. For instance, Perfetti et al. have collected floor and upholstered-seat dust samples from offices and archives of banks, and from headquarters of a highway-company and a media-company in Italy [98]. Detectable Fel d 1 levels were found in 54% of the samples with a low median of 0.025 µg/g. Although the allergen levels were not defined according to the vacuumed surface, the highest Fel d 1 concentrations were detected in samples from upholstered seats. In contrast to office floors, archives floors had no detectable Fel d 1 levels. This may be due to the fact that compared to office spaces, there is less human presence in archives, and thus a reduction in allergen transfer via clothing. Another study from the United States measured cat allergen in floor dust collected in 92 large public and commercial office buildings [99]. Fel d 1 was detected in all but one of the buildings, and in 94% of the samples with a median of 0.3 µg/g. Allergen levels differed slightly according to floor type, a result that must be considered with caution, since 90% of the sampled surfaces were carpets or rugs. Apart from office settings, pet allergens (Fel d 1 and Can f 1) were found in dust samples collected from bedding and carpets in 20 hotels in Brazil [100], but without significant differences between the two sampling sites for both allergens. In this study, pet allergens were also analyzed according to the different hotel classes (Simple, Economical and Superior), but the comfort level of the hotels did not seem to influence the allergen content. The concentrations of domestic allergens have been also measured in hospitals offering in-patients rehabilitation for allergic diseases [101]. Here, mattress dust was collected at two different time points (November and August). Overall, the concentrations of Fel d 1 did not differ among the three sampled hospitals and were significantly higher in autumn than in summer. This seasonal difference in allergen levels may be due to the type of clothing, which, as already discussed, is recognized as the most important vehicle for allergen transfer.

### 4.3. Public Transportation

A large part of the population spends more than 5% of their day inside public transport vehicles. Therefore, allergens in these settings may induce allergic symptoms in susceptible individuals. Consistent with public buildings, very few studies thus far have investigated the occurrence of animal allergens in public transport vehicles (Table 2). One study from Finland measured cat and dog allergen levels in seats and floor dust collected from Helsinki City Transport buses, trams, and underground trains [102]. Both allergens were found in all dust samples, and the concentrations of Can f 1 and Fel d 1 were approximately 10–100 times higher in the dust collected from seats than in dust from floors. Additionally, the authors of this study interviewed passengers to determine how the allergens affected passengers with allergy and asthma. Of the 2021 persons interviewed, 14% reported that pets on public transport caused some inconvenience, usually in the form of health problems. Among passengers with allergy or asthma, 53% reported that they experienced allergic symptoms in public transport, and 11% attributed these symptoms to animals. The widespread distribution of cat allergen in different kind of public transport vehicles is further demonstrated by Pereira et al. [103]. Fel d 1 was measured in 540 dust samples from upholstered seats in 60 buses with air conditioning system, 60 natural ventilation buses and 60 taxis. There were no significant differences in Fel d 1 levels between the three types of vehicles. In taxis, the concentrations of Fel d 1 were significantly higher in the passenger’s rather than in driver’s seats, presumably due to elevated fluctuation of persons. A recent study from Sweden measured cat, dog and horse allergens in the cabin of a commercial aircraft [104]. Samples were collected from two European airline companies (9 airplanes from each company), one with cabins fitted with textile seats (TSC) and another with cabins containing leather seats (LSC). Can f 1, Fel d 1 and Equ c 4 levels were 27 times, 50 times and 75 times higher, respectively in cabins with TSC compared to those with LSC. Cabins are usually cleaned between flights at the airport; however, only the floors are cleaned, which enables allergens to accumulate in the seats, especially in the textile material.

## 5. Conclusions

In addition to residential settings, allergen exposure to animal allergens occurs in a wide range of indoor environments, including workplaces, such as cattle farms and laboratory animal facilities, educational facilities, a variety of public buildings, as well as different modes of public transportation. The ubiquitous spread of animal allergens can be a risk factor, particularly for those already sensitized or symptomatic individuals. Allergen avoidance, which is the first line of prevention against developing allergic symptoms, may be difficult for these patients.

Although the measured values of allergen concentrations are often not directly comparable between the studies due to differences in dust collection method, type of quantification assay and data analysis, many studies show similar effects. The allergen concentrations in a particular environment show a high variability, and are dependent on several environmental factors. Allergen levels can be influenced by pets in and around the environment, the number of pet owners, geographic location, building characteristics, season, humidity and the presence of furniture and carpeting. 

The relationship between allergen exposure to animal allergens (especially cats and dogs) and IgE sensitization or the development of allergic symptoms has been investigated in a number of studies. So far, the results are inconsistent. While many studies found a positive correlation between allergen exposure and incidence of allergies, this association could not be confirmed in other studies. Some studies even showed that high exposure to allergens can have a protective effect against IgE sensitization and allergies. A possible reason for these contradictory findings could be that nearly all studies examined the allergen exposure in one particular environment, mostly at home. Levels of animal allergens that additionally occur in other environments contribute substantially to the total exposure and should be further considered in epidemiological studies.

## Figures and Tables

**Table 1:** Characterized inhalant mammalian allergens (listed in the WHO/IUIS allergen database www.allergen.org).

Animal	Major Allergens	Protein Family	Main Source	MW ^§^ (kDa)	Sensitization Rate ^#^ [Reference]	Other Allergens
**Cat**	Fel d 1 *	Secretoglobin	Saliva, dander	18	84–100% [26,27,28,29,30]	Fel d 2 * (albumin)
	Fel d 4 *	Lipocalin	Saliva	22	31–63% [27,28,29,30,31]	Fel d 3 (cystatin)
						Fel d 5 (IgA)
						Fel d 6 (IgM)
						Fel d 7 (lipocalin)
						Fel d 8 (latherin)
**Dog**	Can f 1 *	Lipocalin	Saliva, dander	23–25	40–76% [27,28,29,30,32]	Can f 2 * (lipocalin)
	Can f 5 *	Kallikrein	Urine	28	31–71% [27,28,29,33]	Can f 3 * (albumin)
						Can f 4 (lipocalin)
						Can f 6 (lipocalin)
						Can f 7 (NPC2 protein)
**Horse**	Equ c 1 *	Lipocalin	Dander, saliva	25	51–76% [27,30,34]	Equ c 2 (lipocalin)
	Equ c 4	Latherin	Dander, saliva	17, 20.5	77% [35]	Equ c 3 * (albumin)
**Cow**	Bos d 2	Lipocalin	Dander	20	83–97% [34,36]	Bos d 3 (calcium-binding protein)
						Bos d 6 * (albumin)
**Mouse**	Mus m 1 *	Lipocalin	Urine	17	66% [34]	
**Rat**	Rat n 1	Lipocalin	Urine	17	88% [37]	
**Guinea pig**	Cav p 1	Lipocalin	Dander, saliva	20	70–87% [38]	Cav p 3 (lipocalin)
	Cav p 2	Lipocalin	Saliva, dander	17	65% [39]	Cav p 4 (albumin)
						Cav p 6 (lipocalin)
**Golden hamster**	Mes a 1	Lipocalin	Saliva, urine, fur	20.5; 24; 30	67–83% [40]	
**Siberian hamster**	Phod s 1	Lipocalin	Saliva, urine, fur	18, 21, 23	100% [41]	
**Rabbit**	Ory c 1	Lipocalin	Fur, saliva	17–18	100% [42]	Ory c 4 (lipocalin)
	Ory c 3	Secretoglobin	Fur	19–21	77% [43]	

* Allergens available for component-resolved allergen diagnostics (ImmunoCAP System or Immuno Solid-Phase Allergen Chip (ISAC), Thermo Fisher Scientific, Phadia AB, Uppsala, Sweden). **^§^** Apparent molecular weight (MW) in sodium dodecyl sulfate-polyacrylamide gel electrophoresis (SDS-PAGE). **^#^** Sensitization rates are based on different studies with different patient groups and test systems (immunoblot, crossed immunoelectrophoresis, ELISA, ImmunoCAP).

**Table 2:** Animal allergen levels in public or work places without the presence of animals.

Sampling Place		Sampling Method	Allergen Levels	Positive Samples	Major Results
**Educational facilities**					
Schools, Sweden [83]	Vacuumed dust from furniture and floor (n = 116)		**Equ c 4 in classes with** >12% horse contact: GM 2051 U/g <12% horse contact: GM 880 U/g	Equ c 4 detected in 96% of samples	Positive relationship between horse allergen levels and rate of horse contact
Schools and homes, USA [84]	Vacuumed dust from floor, desks and chairs (schools, n = 443) and from mattress, bedding and floor (homes n = 321)		**Fel d 1:** Schools: M 0.23 µg/g Homes: M 0.06 µg/g **Can f 1:** Schools: M 0.11 µg/g Homes: M < LOD **Mus m 1:** Schools: M 0.90 µg/g Homes: M 0.14 µg/g **Rat n 1:** Schools: M < LOD Homes: M < LOD	**Fel d 1:** Schools: 94.8% Homes: 79.4% **Can f 1:** Schools: 82.6% Homes: 49.8% **Mus m 1:** Schools: 99.5% Homes: 96.0% **Rat n 1:** Schools: 1.4% Homes: 2.2%	Higher levels of allergens in schools compared to homes Very high levels of Mus m 1 compared to pet allergens
Schools and homes, The Netherlands [85]	EDC placed for 8 weeks in classrooms (n = 123) and bedrooms (n = 169)		**Fel d 1:** Classrooms: GM 60.6 ng/m²/week Homes: GM 13.3 ng/m²/week Homes without cats: 5.6 ng/m²/week Homes with cats: 235.7 ng/m²/week **Can f 1:** Classrooms: GM 35.8 ng/m²/week Homes: GM 7.4 ng/m²/week Homes without dogs: 3.9 ng/m²/week Homes with dogs: 163.5 ng/m²/week **Mus m 1:** Classrooms: GM 1.16 ng/m²/week Homes: GM 0.63 ng/m²/week Homes without pets: 0.54 ng/m²/week Homes with pets: 0.73 ng/m²/week	Not stated	Much higher levels of allergens in schools compared to homes without pets Very low levels of Mus m 1 classrooms and in homes without difference between presence of pets
Schools and homes, USA [86]	Vacuumed dust from classrooms (n = 182) and from bedrooms (n = 271)		**Mus m 1 Classrooms** Signs of mice: M 6.11 µg/g No signs of mice: M 1.21 µg/g **Mus m 1 Homes** Signs of mice: M 0.30 µg/g No signs of mice: M 0.03 µg/g	Mus m 1 detectable in 96.8% of school samples and 92.3% home samples	Strong relationship between signs of mouse and higher levels of mouse allergen
Schools, Malaysia [87]	Vacuumed dust from floor, desks and chairs (n = 32)		**Fel d 1:** M < LOD **Can f 1:** not detectable **Equ c 4:** not detectable	**Fel d 1** detectable only in 3 samples (9%)	Low levels of pet allergens in schools in a tropical country
Day care centers, Poland [88]	Vacuumed dust from floors (n = 84) and childrens’ clothes (n = 36)		**Fel d 1:** Classrooms Winter: GM 0.41 µg/g Classrooms Summer: GM 0.25 µg/g Clothes of all children: GM 0.16 µg/g Clothes of cat owners: GM 3.62 µg/g **Can f 1:**Classrooms Winter: GM 1.29 µg/g Classrooms Summer: GM 0.80 µg/g Clothes of all children: GM 2.17 µg/g Clothes of dog owners: GM 59.2 µg/g	Allergens detectable in over 93% of dust samples	Seasonal variation of animal allergens Pet allergen transportation on the children’s clothes
Day care centers, Germany [89]	Vacuumed dust from smooth floor (n = 561), carpet (n = 469) and furniture (n = 330)		**Fel d 1:** Smooth floor: GM 0.12 µg/g Carpet: GM 0.21 µg/g Furniture: GM 0.67 µg/g **Can f 1:** Smooth floor: GM 0.10 µg/g Carpet: GM 0.15 µg/g Furniture: GM 0.33 µg/g	Fel d 1 and Can f 1 detectable in 96% of samples	Allergen levels differ between various surfaces
**Public Buildings**					
Diverse public places, UK [96,97]		Vacuumed dust from seats and floors	**Fel d 1:** Upholstered seats: 14.88 µg/g Carpeted floors: 0.73 µg/g **Can f 1:** Upholstered seats: 9.4 µg/g Carpeted floors: 1.5 µg/g	Fel d 1 and Can f 1 detectable in 100% of samples	Much higher levels of pet allergens in upholstered seats compared to carpeted floors
Offices and archives, Italy [98]		Vacuumed dust from floors and chairs (n = 160)	**Fel d 1:** M 0.025 µg/g	Fel d 1 detectable in 54% of samples	Highest allergen levels in chairs. No detectable allergens in archives
Offices, USA [99]		Vacuumed dust from floors (n = 251)	**Fel d 1:** Carpet: M 0.31 µg/g Smooth floor: M 0.10 µg/g Other surface: M 0.20 µg/g	**Fel d 1:** Carpet: 94% Smooth floor: 78% Other surface: 94%	
Hotels, Brazil [100]		Vacuumed dust from bedding (n = 98) and carpets (n = 42)	**Fel d 1:** Bedding: GM 0.11 µg/g Carpet: GM 0.09 µg/g **Can f 1:** Bedding: GM 0.3 µg/g Carpet: GM 0.3 µg/g	Not stated	Similar pet allergen concentrations between bedding and carpeted floor
Hospitals, Germany [101]		Vacuumed dust from mattresses (autumn, n = 30, summer n = 30)	**Fel d 1** Autumn: 1.38 µg/g Summer: 0.48 µg/g	Not stated	Seasonal variation of animal allergens
**Public transportation**					
Buses and trams, Finland [102]		Vacuumed dust from seats and floors (n = 18)	**Fel d 1:** Seats: M 0.87 µg/g Floors: M 0.01 µg/g **Can f 1:** Seats: M 2.40 µg/g Floors: M 0.20 µg/g	Fel d 1 and Can f 1 detectable in 100% of samples	Much higher levels of pet allergens in seats compared to floors
Buses and taxis, Brazil [103]		Vacuumed dust from upholstered seats (n = 540)	**Fel d 1:** Buses with AC: GM 1.5 µg/g Buses without AC: GM 1.2 µg/g Taxis: GM 1.6 µg/g	Not stated	No differences in allergen levels between different types of vehicles
Airplanes, Sweden [104]		Vacuumed dust from floor and seats (n = 53)	**Fel d 1:** Textile seats cabin: GM 5.36 µg/g Leather seats cabin: GM 0.11 µg/g **Can f 1:** Textile seats cabin: GM 6.07 µg/g Leather seats cabin: GM 0.23 µg/g **Equ c 4:** Textile seats cabin: GM 13.70 U/g Leather seats cabin: GM 0.18 U/g	Not stated	Textile seats are much more contaminated by pet allergens than leather seats

M: median, GM: geometric mean; LOD: limit of detection; AC: air conditioning.
